# Effect of Lard-Derived Diacylglycerol as a Potential Alternative on the Flavor Characteristics of Frankfurters

**DOI:** 10.3390/foods14234059

**Published:** 2025-11-26

**Authors:** Xinxin Zhao, Yunling Jiang, Zixin Luo, Hai Yu, Jiangyu Zhu, Xinyan Peng, Lang Zhang, Qingfeng Ge, Mangang Wu

**Affiliations:** 1 College of Food Science and Engineering, Yangzhou University, Yangzhou 225127, China; xxzhao@yzu.edu.cn (X.Z.); jiangyunling0513@163.com (Y.J.); 15188580010@163.com (Z.L.); yuhai@yzu.edu.cn (H.Y.); 008051@yzu.edu.cn (J.Z.); qfge@yzu.edu.cn (Q.G.); 2 College of Life Science, Yantai University, Yantai 264005, China; pengxinyan2006@ytu.edu.cn; 3 Key Laboratory of Jianghuai Agricultural Product Fine Processing and Resource Utilization, Ministry of Agriculture and Rural Affairs, Anhui Engineering Research Center for High Value Utilization of Characteristic Agricultural Products, College of Tea & Food Science and Technology, Anhui Agricultural University, Hefei 230036, China; zlang0706@163.com

**Keywords:** lard-derived diacylglycerol, frankfurter, fat substitution, flavor characteristic, electronic nose, gas chromatography-mass spectrometry

## Abstract

Partial or total replacement of pork fat with homologous functional oils may meet consumer demand for healthy meat products while preserving their sensory quality. This study investigated the use of lard-derived diacylglycerol (DG) as a fat replacer on the flavor characteristics of frankfurters. The results revealed that substituting pork fat with purified glycerolized lard (PGL) at different levels (25%, 50%, and 100%) increased the water content and water activity, improved the L* and b* values, and protein thermal stability, while decreasing the a* value of frankfurters. Meanwhile, electronic nose results showed that replacing pork fat with PGL affected the aroma of frankfurters. Furthermore, gas chromatography–mass spectrometry detected 50 volatile compounds in all the frankfurters (such as aldehydes, alcohols, terpenes, and aromatic hydrocarbons, etc.). Replacing lard with PGL significantly increased the variety and content of flavor compounds in frankfurters (*p* < 0.05). According to the approximate odor activity values (OAV) > 1 and variable importance in projection (VIP) > 1, the distinct flavor of the frankfurters with different levels of PGL mainly resulted from aldehydes, alcohols, and terpenes. Generally, this study provided a valuable theoretical foundation for substituting fat with lard-derived DG to improve the flavor characteristics of frankfurters.

## 1. Introduction

Frankfurters are one of the most famous emulsified meat products due to their special flavor, high nutritional value, delicious taste, and convenience. Generally, frankfurters contain high levels of animal fat (up to 20–30%), which helps to improve cooking yield and offer unique flavor, desired texture, solid structure, juiciness, and overall acceptability of foods [1]. However, substantial evidence has shown that increased intake of high-fat diets is detrimental to health (especially obesity and cardiovascular disease) because animal fat generally contains higher levels of cholesterol and saturated fatty acids [2,3]. In recent years, numerous studies have focused on the reducing the fat content of frankfurters by either lowering the amount of animal fat or partially replacing animal fat in frankfurters with plant oils, emulsion gels, emulsions, and gum, etc. [4,5,6,7]. Nevertheless, in comparison with full-fat frankfurters, the frankfurters with reduced animal fat had lower consumer acceptability, flavor, and taste (particularly juiciness). Zhao et al. [8] reported that replacing fat with porcine plasma protein–xanthan gum-based oleogels (PXOs) clearly reduced the flavor and interior color of frankfurters. Botella-Martínez et al. [9] also reported that the high-proportion replacement of pork backfat with gelled emulsions prepared from hemp oil and buckwheat flour tends to disrupt the original flavor balance of the products. Therefore, there is an urgent need to identify healthy alternatives from homologous fat to traditional animal fat.

Animal-derived functional fats, which have similar flavor and sensory properties to animal fat and provide health benefits and nutritional properties and without affecting the fat content, seem to be a suitable fat substitute. As a functional lipid, diacylglycerol (DG) has a similar flavor, taste, and texture to homologous triacylglycerol (TG, the major component of fat) [10]. Meanwhile, it has been demonstrated that DG can reduce the health risks associated with animal fat by inhibiting postprandial serum TG elevation and body fat accumulation. These health benefits of DG are mainly due to the different metabolic pathways of the fats after the absorption of DG and TG [11]. Moreover, DG possesses excellent emulsifying ability and surface activity, which can improve the quality of meat products [12]. Diao et al. [13] investigated the impact of replacing of lard (animal fat) with lard-based DG in emulsion-type sausages and found that DG can enhance the quality and sensory characteristics of sausages. Similarly, our previous studies have shown that lard (pork fat) can be used to prepare healthier lard-derived DG by lipase-catalyzed glycerolysis, and that the addition of DG can produce a compact three-dimensional gel network with enhanced gel strength of pork myofibrillar protein [14,15]. It seems that pork fat DG (lard-derived DG) has emerged as a potential alternative to pork fat. Our previous studies showed that the physicochemical properties, texture, juiciness, flavor intensity, and other sensory properties of frankfurters containing lard-derived DG were superior to those of frankfurters containing lard [16]. Additionally, fat greatly affects the flavor development of cooked meat products via lipid oxidation reactions, and it can influence flavor retention in foods by acting as a solvent for flavor compounds, since many flavor compounds are lipophilic [12,17,18]. The thermal oxidation of fat during cooking usually brings out the desired and delicious flavor of meat products [19]. Fat also affects flavor release by reducing the vapor pressure of lipophilic compounds [20]. However, information on the effect of DG on the flavor profile of frankfurters remains limited.

Hence, the present study aimed to investigate the effect of lard-derived DG on the flavor attributes of frankfurters using gas chromatography-mass spectrometry (GC-MS) combined with electronic nose. Comparisons of the water content, water activity, pH, color, and thermal property of frankfurters containing different levels of DG were also carried out. This study provides information on the flavor profile of frankfurters with different levels of lard-derived DG and offers guidelines for the producing healthier and more flavorful frankfurters by using lard-derived DG as an alternative to pork fat.

## 2. Materials and Methods

### 2.1. Preparation of Lard-Derived Diacylglycerol

Lard-derived diacylglycerol (DG) was prepared using our previously reported method [14]. Briefly, pork backfat was purchased from Sushi Meat Products Co., Ltd. (Huai’an, Jiangsu, China), heated to 120 °C with constant stirring, and then filtered to obtain liquid lard. The liquid lard was then mixed with glycerol at a molar ratio of 1:1, and Lipozyme^®^ RM [with an enzyme activity of 275 Inter Esterase Units Novo (IUN)/g, purchased from Novozymes A/S Co., Ltd. (Bagsvaerd, Denmark)] was added at a ratio of 4:100 (*w*/*w*, enzyme to lard). Then, the mixture was ultrasonicated for 5 min at 250 W and 45 °C using an XH-2008D ultrasonic instrument (Xianghu Development Co., Ltd., Beijing, China). After ultrasonication, the mixture was maintained in a shaking water bath at 50 °C for 4 h, then filtered to collect the sample. To further increase the DG content, two-step SPE10 molecular distillation (Haiyuan Biochemical Equipment Co., Ltd., Wuxi, China) was used for purification. In the first step, the evaporation temperature and vacuum were set at 205 °C and 60 Pa, respectively. In the second step, the evaporation temperature and vacuum were set at 280 °C and 22 Pa, respectively. The DG content in the purified glycerolized lard (PGL) was determined to be 83.10% using high-performance liquid chromatography.

### 2.2. Preparation of Frankfurters

The lean pork and back fat were both supplied by Yangzhou Sushi Meat Products Co., Ltd. (Yangzhou, Jiangsu, China). Three independent batches of frankfurters were prepared, with each batch containing four treatment groups, and each treatment containing 20 frankfurters (approximately 85 g of each frankfurter). The formulations for the four different types of frankfurters were shown in Table 1 and were the identical except for the fats. These fats were lard substituted by PGL at different levels (0%, 25%, 50%, and 100%). The frankfurters were manufactured using the procedure described by Zhao et al. [16]. Finally, the cooked frankfurters were vacuum packed and stored at 4 °C until further analysis.

### 2.3. Water Content, Water Activity and pH

Water content was detected by the constant weight method. Briefly, the frankfurters were dried in an oven at 105 °C and then weighed to calculate the water content. Water activity (a_w_) was measured directly using an AquaLab 4TE DUO water activity meter (Decagon Devices, Pullman, WA, USA). The pH of the frankfurters was determined with a DELTA 320 pH meter (Mettler-Toledo GmbH, Greifensee, Switzerland) according to the method of Li et al. [21].

### 2.4. Color Measurement

After the frankfurters were cooled at 25 °C for 30 min, the lightness value (L*), redness value (a*) and yellowness value (b*) of the sample surface were measured with a ZE-6000 colorimeter (Nippon Denshoku, Kogyo Co., Tokyo,  Japan) with a D65 illuminant, a 10° standard observer, and a 10 mm aperture.

### 2.5. Differential Scanning Calorimetry

The thermal stability of raw frankfurter batters (Section 2.2 without drying, smoking, and cooking) was analyzed by differential scanning calorimetry (DSC, TA Instruments Inc.,  New Castle, DE, USA) under a nitrogen atmosphere according to the method described by Sun et al. [22]. The peak temperature (*T*_max_) and enthalpy value (Δ*H*) were recorded.

### 2.6. Electronic Nose Analysis

The electronic nose analysis of the frankfurters was analyzed using a PEN3 electronic nose (Airsence, Schwerin, Germany) as described by Cao et al. [23]. The electronic nose system contains 10 metal oxide semiconductor (MOS) sensors, each specifically recognizing different volatile compounds, as shown in Table 2.

### 2.7. Gas Chromatography-Mass Spectrometry Analysis

The gas chromatography-mass spectrometry (GC-MS) analysis of the frankfurters was performed as described by Yuan et al. [24] with slight modifications. Chopped sample (3 g) was placed in a 20 mL sealed headspace vial (CNW Technologies, Düsseldorf, Germany) and mixed with 2 μL of internal standard 1,2-dichlorobenzene (100 mg/L in methanol). After equilibrating in a water bath at 45 °C for 25 min, a solid phase microextraction (SPME) fiber coated with divinylbenzene/carboxen/polydimethylsiloxane (DVB/CAR/PDMS) (50/30 μm, Sigma Aldrich Supelco Corp., Bellefonte, PA, USA) was inserted into the headspace vial and extracted for 45 min. After extraction, the fiber was immediately transferred and inserted into the injection port and desorbed at 230 °C for 3 min.

Volatile compounds were analyzed using a GCMS-QP2020 NX gas chromatography-mass spectrometry (GC-MS) system (Shimadzu Co., Kyoto, Japan) with an Inert Cap Wax (0.25 mm × 0.25 μm × 60 m) capillary column. The column temperature was initially held at 40 °C for 3 min, then heated to 200 °C at a rate of 5 °C/min, and ultimately raised to 230 °C at a rate of 10 °C/min and kept for 2 min. The flow rate of the carrier gas (helium) was 1 mL/min. The ion source temperature was 230 °C, and the mass spectrometer scanned masses over the range *m*/*z* 45–500. The volatile compounds in the frankfurters were identified by comparing the mass spectra to a mass spectra library from NIST 17. Semiquantification of the volatile compounds was performed by dividing the peak area of the target compounds by the peak area of the internal standard and multiplying by the initial content of the internal standard (expressed as μg/kg).

The odor activity value (OAV) represents the contribution of the volatile compound to flavor. The OAV of volatile compounds in frankfurters is calculated by dividing their relative content in the frankfurter by their odor threshold in water [25]. The reference odor threshold values come from the book [26].

### 2.8. Statistical Analysis

Three independent batches of frankfurters (replicates) were prepared on different days. Each test was carried out at least three times. The results were expressed as mean ± standard error (SE). The data were analyzed by one-way analysis of variance (ANOVA) using the General Linear Model procedure in the Statistix 8.1 software package, and the significant differences (*p* < 0.05) were determined by Tukey’s procedures. Linear discriminant analysis (LDA) of the electronic nose was generated using the matching WinMuster software  (version 1.6.2.18, Airsense Analytics GmbH, Schwerin, Germany). The hierarchical cluster analysis heat map was generated using TBtools software (version 2.012). The data were normalized prior to plotting. Partial least squares discriminant analysis (PLS-DA) of the GC-MS was analyzed using the SIMCA software (version 14.1, Umetrics, Umea, Sweden). Prior to the PLS-DA analysis, the data were pre-treated using mean-centering and unit variance scaling in SIMCA software (version 14.1). All other plots were performed using Origin (version 2021, Originlab Co., Northampton, MA, USA).

## 3. Results and Discussion

### 3.1. Water Content, Water Activity and pH

As shown in Table 3, the water content and water activity (a_w_) of the 100% Lard group were the lowest (48.88% and 0.96, respectively). The addition of DG oils significantly increased the water content and water activity (*p* < 0.05), and with the increase in the substitution ratio of PGL, the water content gradually increased (*p* < 0.05), but the water activity had no significant change (*p* > 0.05). The water content and water activity of the 100% PGL group were the highest (60.89% and 0.97, respectively), and there was no significant difference between the 50% PGL and 100% PGL groups (*p* > 0.05). The probable reason for this is that DG oils with one hydroxyl group are more hydrophilic than TG oils [11]. The increase in water content and a_w_ with the addition of DG oils was also reported by Miklos et al. [27], who found that fermented sausages with DG had significantly higher a_w_ (0.794) and water content (31.02%) compared to the fermented sausages with backfat (a_w_ and water content were 0.786 and 30.53%, respectively) (*p* < 0.05). Mora-Gallego et al. [28] also reported that the a_w_ of non-acid fermented sausages with DG was significantly higher than that of non-acid fermented sausages with backfat (*p* < 0.05). The results for water content and water activity (a_w_) were consistent with those for cooking loss and emulsion stability above. In addition, no significant difference in pH was observed between the different treatments, suggesting that the type of fat did not affect the pH of the frankfurters.

### 3.2. Color Measurement

Color is an important indicator in assessing the quality of meat products because it influences consumer choice and product appeal, which is related to the type and amount of fat used [29]. As shown in Table 4, compared with the 100% Lard group, the L* value and b* value of the 25% PGL group, 50% PGL and 100% PGL group were significantly increased (*p* < 0.05), while the a* value was significantly decreased (*p* < 0.05), with no significant difference among the three groups (*p* > 0.05). This result indicated that the addition of DG oils to replace pork fat affected the internal color of frankfurters, which may be related to the smaller particle size and larger surface area of DG oils, resulting in greater light reflection, rather than being caused by the type of fat used (TG oil and DG oil).

### 3.3. Differential Scanning Calorimetry

DSC is often used to study the protein thermal stability of meat products. The peak temperature (*T*_max_) and enthalpy value (Δ*H*) indicate the protein denaturation temperature and the energy required to denature proteins, respectively [22]. As shown in Table 5, all frankfurter treatments had two typical transition peak temperatures at 50.09–53.60 °C (*T*_max1_) and 69.91–76.75 °C (*T*_max2_), which correspond to the denaturation temperatures of myosin and actin [22]. Similarly, Zhou et al. [30] also found two major transition peaks at 54.40–58.48 °C and 67.57–70.28 °C in all pork sausage treatments. Both the transition peak temperatures and enthalpy values of the two peaks increased with increasing the substitution ratio of PGL. For example, the transition peak temperatures (*T*_max1_ and *T*_max2_) and enthalpy values (Δ*H*_1_ and Δ*H*_2_) of the 50% PGL and 100% PGL groups were significantly higher than those of the 100% lard and 25% PGL groups (*p* < 0.05). However, there was no significant difference in the transition peak temperatures and enthalpy values between the 50% PGL and 100% PGL groups. These results indicated that the addition of DG improved the thermal stability of muscle proteins due to the occurrence of interaction between muscle proteins and DG, resulting in the formation of complexes with dense and ordered structures.

### 3.4. Electronic Nose Analysis

**Figure 1:**
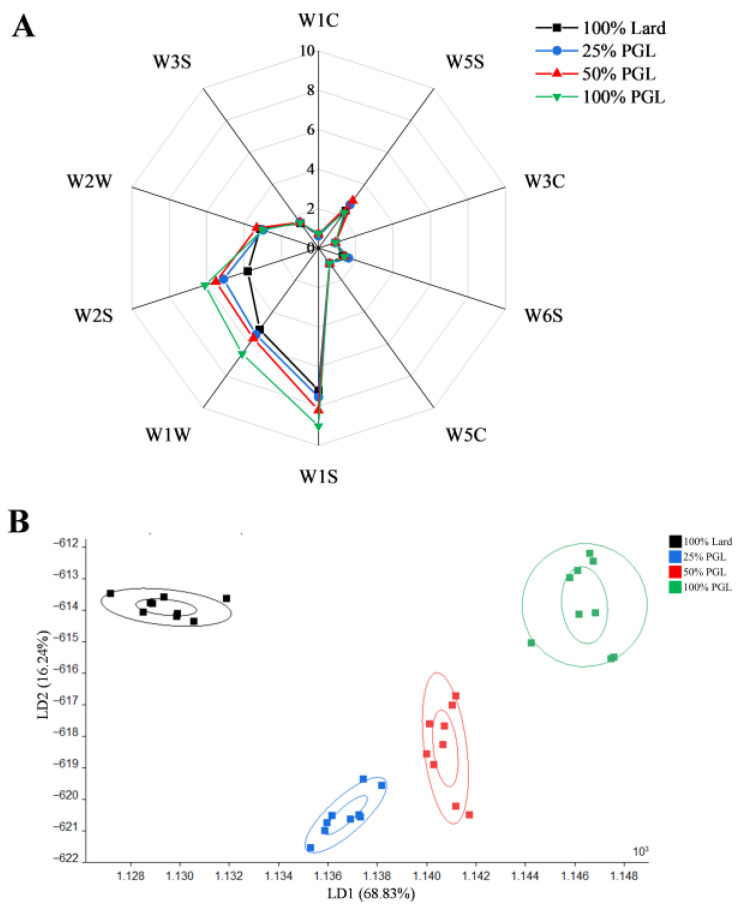
Radar chart (**A**) and linear discriminant analysis score plot (**B**) of the electronic nose data for frankfurters prepared with 100% lard or lard replaced with 25%, 50% or 100% purified glycerolized lard (PGL).

As shown in Figure 1A and Table 2, the W1S, W2S, W5S, W1W, and W3S sensors showed higher responses to the flavor compounds in frankfurters, suggesting that frankfurters had higher abundances of alcohols, aldehydes, ketones, methyl, nitrogen oxides, sulfides compounds, and long-chain alkanes. Compared with the frankfurters with 100% lard, the signal intensities of the W1S, W2S, and W1W sensors were higher for frankfurters with PGL. Notably, the 100% PGL group showed the highest signal intensities, indicating that frankfurters with PGL can produce higher abundances of alcohols, aldehydes, ketones, methyl, and sulfides compounds. These results were also verified by the GC-MS analysis, which indicated that the frankfurters with different amounts of PGL contained higher concentrations of alcohols [e.g., 1-octen-3-ol, (−)-terpinen-4-ol, and linalool], aldehydes (e.g., hexanal and nonanal), and methyl compounds (e.g., α-terpinene, benzene, 1,3-diethyl-5-methyl-, 1-allyl-2-methylbenzene, 2,6-dimethyldecalin, 2-methylindene, 2,2,4,6,6-pentamethylheptane, anethole, etc.). Similarly, Zhao et al. [8] detected methyl, alcohols, aldehydes, ketones, long-chain alkanes, hydrides, and sulfides in frankfurters. Chevance et al. [31] also reported that the aldehydes, alcohols, and sulfur compounds were detected in frankfurters. Our previous study has also indicated that replacing lard with PGL improved the sensory characteristics of the frankfurters, with those containing 100% PGL having the highest flavor intensity scores [16].

To investigate the differentiation between frankfurters with different substitution ratios of PGL, linear discriminant analysis (LDA) was performed based on the electronic nose data. The frankfurter matrix was kept constant, and the only source of variability was the percentage of lard replaced with PGL. As shown in Figure 1B, the first two LDs accounted for 85.07% of the total variance (LD1: 68.83%, LD2: 16.24%), indicating that these two LDs can explain the majority of flavor information of the frankfurters. The difference between the samples is positively correlated with the distance between the samples on the X-axis. Four groups were obviously separated and located in different regions with no overlap, indicating that frankfurters containing different levels of PGL had different odors and that LDA could distinguish between the different treatments. The relative distance reflects the difference in overall odor between the frankfurters, with greater distances indicating larger odor differences. The largest relative distance was found between the 100% Lard group and the other groups with different PGL replacement ratios. As the PGL replacement ratio increased, the relative distance between the 100% Lard group and the groups with different substitution ratios increased, with the 100% PGL group having the largest relative distance from the 100% Lard group. This suggested that the replacement of lard with PGL altered the flavor of frankfurters, with larger replacement ratios leading to more significant alterations in overall odor. Similarly, Wu et al. [32] reported that LDA was an effective way of discriminating the flavors of the stewed bone-in lamb loin.

### 3.5. Gas Chromatography-Mass Spectrometry Analysis of Volatile Compounds

#### 3.5.1. Volatile Compounds and Odor Activity Value Analysis

Flavor is also an essential factor influencing consumer acceptance of meat products. To understand the flavor attributes of frankfurters, the composition and relative content of volatile compounds were investigated by GC-MS. The types and relative contents of the volatile compounds in frankfurters with different substitution ratios of PGL are shown in Table 6, with a total of 50 volatile compounds classified into seven categories, including 7 aldehydes, 5 alcohols, 15 terpenes, 14 aromatic hydrocarbons, 3 acids, 2 esters, and 4 others. The composition and relative content of volatile compounds in the frankfurters were affected by PGL substitution. A total of 32, 45, 44, and 41 volatile compounds were detected in the 100% Lard, 25% PGL, 50% PGL, and 100% PGL groups, respectively. Moreover, the replacement of lard with DG increased the content of most volatile compounds, with the 100% PGL group showing the greatest abundance. These results indicated that replacing pork fat with DG oils was conducive to the generation of volatile compounds in frankfurters, which can be explained by the lipid oxidation. Lipid and/or fat influenced flavor development due to lipid oxidation, which led to the formation of flavor compounds such as aldehydes, alcohols, and esters, resulting in the unique aroma of meat products [33]. Li et al. [21] reported that thiobarbituric acid-reactive substance (TBARS) levels in 100% PGL-based frankfurters were higher than those in 100% lard-based frankfurters. Lipid and/or fat not only influenced flavor retention behaviors because of the solubilization of liposoluble volatile compounds in it, but also affected flavor release by reducing the vapor pressure of lipophilic compounds [20].

Aldehydes predominantly originate from lipid oxidation (especially the degradation of unsaturated FAs) and obviously influence the animal species-specific meat flavor due to their high relative contents and low odor threshold [24]. Hexanal was the most abundant aldehyde compound found in frankfurters, and its content was found to be linked with lipid oxidation [34]. Meanwhile, the relative content of aldehydes in frankfurters containing PGL exceeded that in frankfurters containing 100% lard (*p* < 0.05), and increased with increasing PGL content (*p* < 0.05), which was in line with the results in the electronic nose. Flavor perception is not only related to the content of flavor compounds, but also depends on the threshold values of these flavor compounds [26]. Odor activity value (OAV) reflects the contribution of flavor compounds to aroma and is calculated by the ratio of the content to the odor threshold of the flavor compounds [35,36]. Generally, flavor compounds with OAV > 1 are regarded as key aroma-active compounds that contribute to the flavor characteristics. The approximate OAV of three aldehydes (hexanal, octanal, and nonanal) was greater than 1 (Table 7). This phenomenon is due to the high relative content and low odor threshold of these aldehydes, which play an important role in the overall odor of frankfurters. Chevance et al. [31] reported that aldehydes were derived from the meat fraction in frankfurters, with hexanal contributing to the green odor. This may be because the 100% Lard group showed the highest cooking loss and the lowest emulsion stability [16], thereby resulting in the highest total released liquid and released fat, which increases the loss of volatile compounds via the juice. Similarly, Yuan et al. [24] reported that frankfurters with the highest cooking yield had significantly higher content of aldehydes. High temperature also promotes the lipid oxidation to produce aldehydes, especially in frankfurters containing PGL that have undergone purification at high temperature. Meanwhile, the viscosity of DG (84.8 mPa·s) is higher than that of TGs (74.5 mPa·s) [37]. The higher the viscosity of the food matrix, the stronger the adsorption effect on volatile compounds [38], which is another possible reason for the higher content of aldehydes of frankfurters containing PGL.

Alcohols primarily originate from lipid oxidation and spices [39], with 5 alcohols being detected in all treatments. The 100% Lard group had the lowest relative content of alcohols (*p* < 0.05), particularly 1-hexanol, 1-octen-3-ol, linalool, and (−)-terpinen-4-ol, which were significantly lower than in frankfurters with PGL (*p* < 0.05), implying that the addition of DG increased the relative content of alcohols. Based on OAV, the main contributors of alcohols to frankfurters were 1-hexanol, 1-octen-3-ol, and linalool. As reported by Wang et al. [40], alcohols were likely to bind to muscle proteins owing to the number of hydrogen bonds present in alcohols. Meanwhile, the presence of a hydrogen bond in DG, and thus the addition of PGL, resulted in an increase in hydrogen bonds. Alcohols interact with muscle proteins through hydrogen bonds, which reduces the volatilization of volatile flavor compounds during the heating [24]. Additionally, some volatile compounds, such as linalool and α-terpineol, may originate from the spice (coriander seed) used in formulation [41].

Terpenes possess a spicy aroma and are mainly produced by the addition of spices (such as white pepper powder, ground ginger powder, ground nutmeg powder, bell pepper powder, and ground coriander seed powder) to frankfurters. For instance, γ-terpinene and 3-carene mainly originate from coriander seed and white pepper, respectively [25,41,42]. β-Pinene has been confirmed in nutmeg and coriander seed [31,41]. In this study, terpenes were the most diverse class of volatile compounds in sausages, with a total of 15 terpenes being identified. In particular, γ-terpinene was the most abundant terpene in frankfurters. The relative content of terpenes in the 100% lard group was significantly lower than in the 25% PGL, 50% PGL, and 100% PGL groups (*p* < 0.05), indicating that adding a higher DG content has a positive impact on the production and release of terpenes. Meanwhile, the main contributor to the frankfurters odor from terpenes was (+)-dipentene based on its higher approximate OAV. It has been speculated that the greater juice loss in the 100% Lard group caused the terpenes to escape via the juice. Zhao et al. [16] reported that the juiciness score of frankfurters increased as the PGL content increased, with frankfurters containing 100% lard achieving the lowest score. Another possible reason is that emulsions prepared with DG have smaller droplets than those prepared with TG. Charles et al. [43] reported that reducing the size of the droplets in emulsions increased the release of volatile compounds in salad dressings.

Aromatic hydrocarbons were the most abundant in frankfurters with PGL (few types of aromatic hydrocarbons were detected in the control group) and possibly originate from the thermal oxidation of lipids [25,44]. This is possibly because the PGL preparation undergoes a two-step molecular distillation purification process at high temperatures (205 and 280 °C). Diao et al. [45] also reported that the heating process involved in DG synthesis. Long-chain fatty acids present in fats undergo auto- and thermal oxidation (via peroxide-initiated free radical decomposition) to form long-chain unsaturated hydrocarbons, which then form aromatic hydrocarbons (such as alkylbenzenes). Watanabe et al. [46] reported that benzene, xylenes, isopropylbenzene, and several uncharacterized C_4_–C_6_ alkylbenzenes were detected in fried beef fat at high temperature (cooking temperature of 145 °C). However, aromatic hydrocarbons have high odor thresholds in water. For instance, the odor thresholds of m-cymene, 1,3,5-triethylbenzene, and 2-pentylfuran are 800, 170, and 14.5 μg/kg, respectively [47,48,49]. Furthermore, the majority of volatile compounds are hydrophobic and have a stronger affinity for oils, as well as a higher odor threshold in oil than in water [50]. Therefore, aromatic hydrocarbons contribute very little to the flavor characteristics of frankfurters. Similarly, Chevance et al. [31] reported that the contribution of the aromatic hydrocarbons to frankfurters was smaller than that of the aldehydes and alcohols.

Additionally, safrole and myristicin are generated from spice (nutmeg), and their relative content increased with increasing PGL addition levels. This result indicated that adding DG may promote the release of safrole and myristicin due to their greater solubility and lipophilicity in lipids than in water. Meanwhile, DG is less hydrophobic than TG due to the presence of a hydrophilic hydroxyl group in DG that replaces a hydrophobic fatty acid chain. Therefore, in foods containing lipids, more volatile compounds are released from the lipid phase into the aqueous phase, where they mainly interact with proteins through hydrophobic interactions [51,52].

#### 3.5.2. Principal Component Analysis and Clustering Visualization

**Figure 2:**
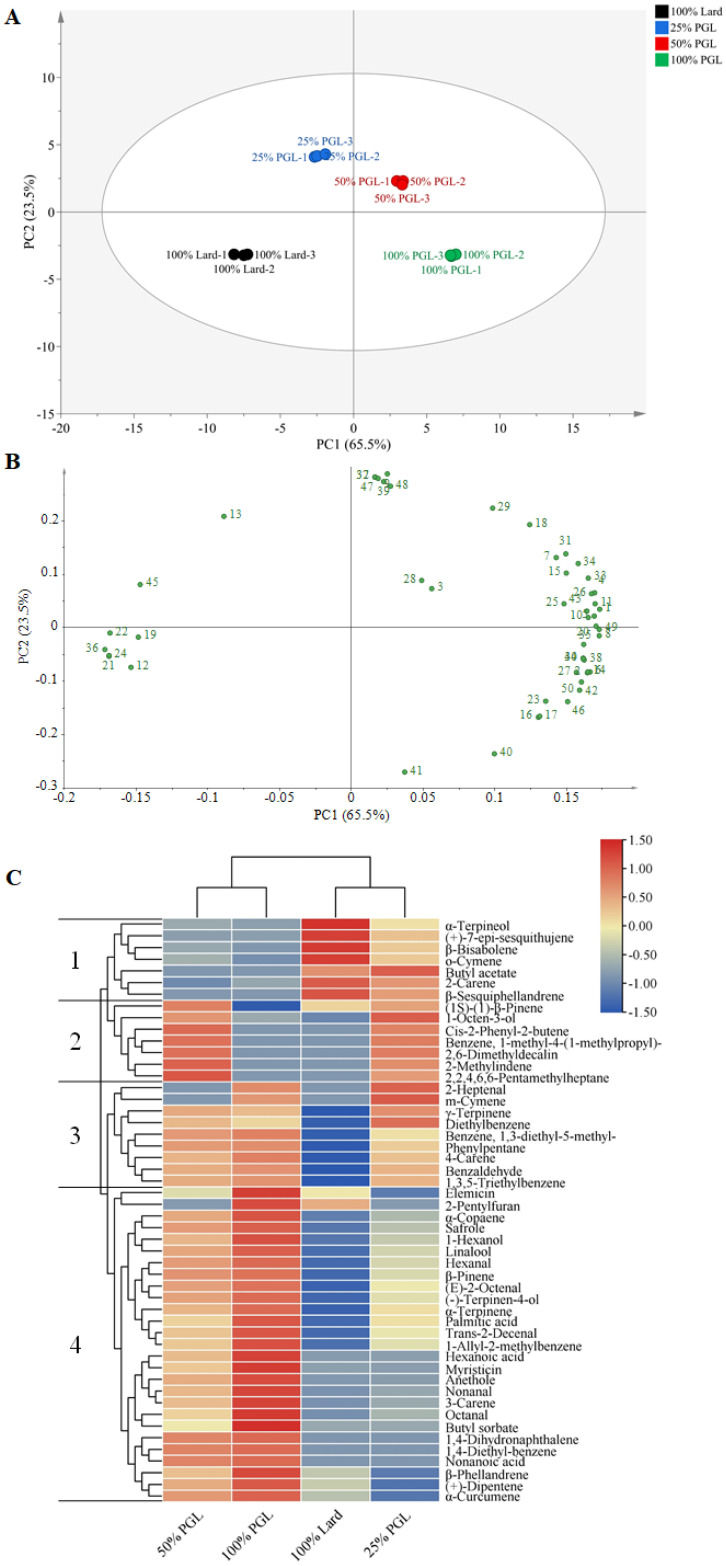
Principal component analysis score plot (**A**), loading plot (**B**) and hierarchical cluster analysis heat map (**C**) of volatile compounds for frankfurters prepared with 100% lard or lard replaced with 25%, 50% or 100% purified glycerolized lard (PGL). The vertical axis represents the sample number, and the horizontal axis represents the detected volatile compounds. The blue and red blocks represent relatively low and high content, respectively.

Principal component analysis (PCA) is a non-supervised chemometric method and an exploratory tool based on dimensionality reduction [53]. The PCA plot enables an overall analysis of the variability within and between the groups of samples. Higher similarity between samples corresponds to a higher degree of clustering, while higher differences between the samples correspond to greater distances [54]. In order to investigate the differences in the flavor profiles of frankfurters containing 100% lard or lard replaced with 25%, 50% or 100% PGL, a PCA was carried out to describe the distribution of the four treatments. As shown in Figure 2A, a total of 89% of the variance was explained by the first two principal components, which was greater than 80%. This indicated that the first two principal components explained the majority of the flavor characteristics of frankfurters [55]. The four treatments were clearly separated with no overlap, indicating that the flavor profile of frankfurters with different levels of PGL was distinctly different. Notably, the 100% Lard group was the furthest from the 100% PGL group on the PC1 axis, demonstrating the most obvious difference in flavor characteristics between the two groups. As shown in Figure 2B, the 50% PGL and 100% PGL groups were found on the positive axis of the PC1, and they were positively correlated with hexanal (No. 1), octanal (No. 2), nonanal (No. 6), 1-hexanol (No. 8), linalool (No. 10), (+)-dipentene (No. 16), 1-allyl-2-methylbenzene (No. 35), hexanoic acid (No. 42), and safrole (No. 49), etc. The 100% Lard and 25% PGL groups were found on the negative axis of the PC1, and they were positively correlated with β-sesquiphellandrene (No. 22), β-bisabolene (No. 24), and o-cymene (No. 36), etc. This indicated that the volatile profiles of the 50% PGL and 100% PGL groups were obviously different from those of the 100% Lard and 25% PGL groups. In other words, the flavor characteristics of frankfurters with a high PGL replacement level were clearly different from those of the 100% Lard and low PGL replacement groups, which coincides with the LDA result of the electronic nose.

Hierarchical cluster analysis (HCA) was employed to identify differences in volatile compounds that play a key role in frankfurters with different levels of PGL. As shown in Figure 2C, in the vertical pattern, the 50% PGL and 100% PGL groups were clustered into one category, and the 100% Lard and 25% PGL groups were clustered into another category. Meanwhile, all volatile compounds were distinguished into four clusters. In zone 1, (+)-7-epi-sesquithujene, β-bisabolene, o-cymene, butyl acetate, 2-carene, and β-sesquiphellandrene increased in the 100% Lard and 25% PGL groups. In zone 2, 1-octen-3-ol, cis-2-phenyl-2-butene, benzene, 1-methyl-4-(1-methylpropyl)-, 2,6-dimethyldecalin, 2-methylindene, and 2,2,4,6,6-pentamethylheptane increased in the 25% PGL and 50% PGL groups. In zone 3, γ-terpinene, 4-carene, benzaldehyde, and 1,3,5-triethylbenzene increased in the 25% PGL, 50% PGL, and 100% PGL groups. In zone 4, 1,4-dihydronaphthalene, 1,4-diethyl-benzene, nonanoic acid, α-copaene, safrole, 1-hexanol, linalool, β-pinene, (E)-2-octenal, (−)-terpinen-4-ol, α-terpinene, anethole, nonanal, 3-carene, (+)-dipentene, β-phellandrene, and α-copaene increased in the 50% PGL and 100% PGL groups. These results showed that the addition of PGL could markedly influence the flavor profile of frankfurters, which was in line with the results of GC-MS and PCA. Our previous studies also showed that the addition of DG (the main component of PGL) increased the flavor intensity score of frankfurters [15].

#### 3.5.3. Partial Least Squares Discriminant Analysis

**Figure 3:**
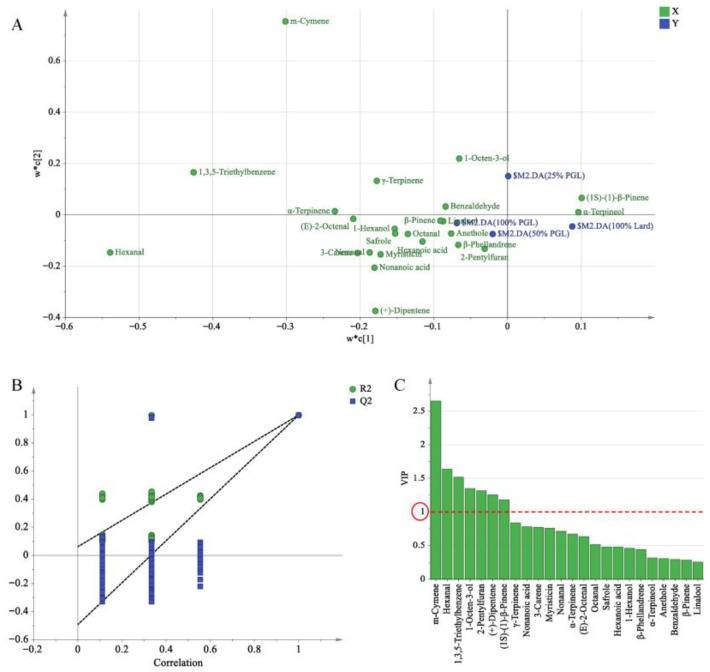
Partial least squares discriminant analysis (PLS-DA) based on approximate odor activity values (OAV) data of volatile compounds for frankfurters prepared with 100% lard or lard replaced with 25%, 50% or 100% purified glycerolized lard (PGL): (**A**) loading scatter plot, (**B**) permutation test plot, (**C**) variable importance in projection (VIP) plot.

Partial least squares discriminant analysis (PLS-DA) was used to identify the volatile compounds based on OAV data responsible for the differences in frankfurters containing different levels of PGL. As shown in Figure 3A, R^2^X, R^2^Y, and the prediction ability parameter Q^2^ were 0.969, 0.998, and 0.995, respectively, all of which were close to 1. This showed that the model possessed good interpretation and classification predictive ability. Meanwhile, the model was not overfitting because the intercept of the Q^2^ regression line on the Y-axis was less than zero (Figure 3B). To confirm the main variables causing the PLS-DA model classification, the contribution of volatile compounds was analyzed using variable importance in projection (VIP) based on PLS-DA. In general, VIP > 1 indicates that the variables significantly affect the sample classification [56]. As shown in Figure 3C, 7 volatile compounds with VIP > 1 were detected, including m-cymene (No. 28), hexanal (No. 1), 1,3,5-triethylbenzene (No. 31), 1-octen-3-ol (No. 9), 2-pentylfuran (No. 41), (+)-dipentene (No. 16), and (1S)-(1)-β-pinene (No. 13).

According to the OAV > 1 and VIP > 1, 3 volatile compounds (including 1 aldehyde, 1 alcohol, and 1 terpene) played a vital role in the flavor profile of frankfurters with varying levels of PGL. The above volatile compounds [hexanal, 1-octen-3-ol, and (+)-dipentene] could be used as key volatile compounds and markers to differentiate between the frankfurters with different levels of PGL.

## 5. Conclusions

In conclusion, this study identified 50 volatile compounds, 7 of which were found to play a pivotal role in the overall flavor profile of frankfurters based on OAV > 1. Replacing lard with PGL increased the variety and content of volatile compounds in frankfurters. Furthermore, aldehydes (hexanal), alcohols (1-octen-3-ol), and terpenes [(+)-dipentene] could be regarded as the key volatile compounds that distinguished frankfurters with different levels of PGL according to the OAV (>1) and VIP (>1) results. Additionally, DG improved the quality of frankfurters, which is specifically reflected in the increase in L*- and b*-values, water content and water activity, and protein thermal stability. Hence, lard-derived DG may be a promising fat substitute for producing healthy and high-quality meat products. Future studies could focus on intelligent sensory techniques, such as the electronic tongue technology and gas chromatography-olfactometry (GC-O).

## Figures and Tables

**Table 1:** The formulations of frankfurters prepared with 100% lard or lard replaced with 25%, 50% or 100% purified glycerolized lard (PGL).

	100% Lard	25% PGL	50% PGL	100% PGL
Lean meat (g)	1000	1000	1000	1000
Lard (g)	500	375	250	0
PGL (g)	0	125	250	500
Ice (g)	500	500	500	500
Salt (g)	30	30	30	30
Composite phosphate (g)	8	8	8	8
Sodium nitrite (g)	0.15	0.15	0.15	0.15
Monosodium glutamate (g)	1	1	1	1
Sodium erythorbate (g)	2	2	2	2
Soy protein isolate (g)	30	30	30	30
White pepper powder (g)	6	6	6	6
Ground ginger powder (g)	6	6	6	6
Ground nutmeg powder (g)	5	5	5	5
Bell pepper powder (g)	5	5	5	5
Ground coriander seed powder (g)	1	1	1	1

**Table 2:** Information of 10 sensors for electronic nose.

Sensors	Representative Substance	Description
W1C	Aromatic compounds	Sensitive to aromatic constituents, benzene
W5S	Broad range	Sensitive to nitrogen oxides
W3C	Aromatic	Sensitive to aroma, ammonia
W6S	Hydrogen	Sensitive to hydrides
W5C	Arom-aliph	Sensitive to short-chain alkane aromatic component
W1S	Broad-methane	Sensitive to methyl
W1W	Sulphur-organic	Sensitive to sulfides
W2S	Broad-alcohol	Sensitive to alcohols, aldehydes and ketones
W2W	Sulph-chlor	Sensitive to organic sulfides
W3S	Methane-aliph	Sensitive to long-chain alkanes

**Table 3:** Water content, water activity and pH of frankfurters prepared with 100% lard or lard replaced with 25%, 50% or 100% purified glycerolized lard (PGL).

Sample	Water Content (%)	a_w_	pH
100% Lard	48.88 ± 1.04 ^c^	0.96 ± 0.01 ^b^	6.38 ± 0.01 ^a^
25% PGL	52.89 ± 0.43 ^b^	0.97 ± 0.01 ^a^	6.40 ± 0.01 ^a^
50% PGL	56.94 ± 0.79 ^a^	0.97 ± 0.01 ^a^	6.41 ± 0.01 ^a^
100% PGL	60.89 ± 0.13 ^a^	0.97 ± 0.01 ^a^	6.42 ± 0.01 ^a^

Values are expressed as the mean ± standard error (SE). Different lowercase letters (a–c) in the same column indicate significant differences (*p* < 0.05).

**Table 4:** Color of frankfurters prepared with 100% lard or lard replaced with 25%, 50% or 100% purified glycerolized lard (PGL).

Sample	L*	a*	b*
100% Lard	57.59 ± 0.54 ^b^	11.74 ± 0.23 ^a^	17.16 ± 0.25 ^b^
25% PGL	60.97 ± 0.48 ^a^	10.57 ± 0.23 ^b^	18.19 ± 0.05 ^a^
50% PGL	61.89 ± 1.07 ^a^	10.03 ± 0.28 ^b^	18.45 ± 0.14 ^a^
100% PGL	62.53 ± 1.01 ^a^	9.48 ± 0.35 ^b^	18.79 ± 0.25 ^a^

Values are expressed as the mean ± standard error (SE). Different lowercase letters (a,b) in the same column indicate significant differences (*p* < 0.05).

**Table 5:** Differential scanning calorimetry (DSC) analysis of frankfurters prepared with 100% lard or lard replaced with 25%, 50% or 100% purified glycerolized lard (PGL).

	Transition Temperature (°C)		Enthalpy (J/g)	
	*T*_max1_	*T*_max2_	Δ*H*_1_	Δ*H*_2_
100% Lard	50.09 ± 0.21 ^c^	69.91 ± 0.40 ^c^	0.76 ± 0.04 ^b^	0.16 ± 0.02 ^c^
25% PGL	51.66 ± 0.20 ^b^	72.86 ± 0.40 ^b^	1.81 ± 0.11 ^b^	0.43 ± 0.02 ^b^
50% PGL	53.18 ± 0.17 ^a^	75.36 ± 0.30 ^a^	4.54 ± 0.18 ^a^	0.74 ± 0.04 ^a^
100% PGL	53.60 ± 0.36 ^a^	76.75 ± 0.40 ^a^	5.83 ± 0.40 ^a^	0.97 ± 0.07 ^a^

Values are expressed as the mean ± standard error (SE). Different lowercase letters (a–c) in the same column indicate significant differences (*p* < 0.05).

**Table 6:** Relative contents (μg/kg) of volatile compounds in frankfurters prepared with 100% lard or lard replaced with 25%, 50% or 100% purified glycerolized lard (PGL).

No	Volatile Compounds	CAS	100% Lard	25% PGL	50% PGL	100% PGL
	**Aldehydes**					
1	Hexanal	66-25-1	46.37 ± 0.74 ^d^	83.27 ± 0.78 ^c^	111.58 ± 0.88 ^b^	124.92 ± 1.72 ^a^
2	Octanal	124-13-0	6.12 ± 0.27 ^d^	7.09 ± 0.09 ^c^	8.92 ± 0.17 ^b^	11.94 ± 0.08 ^a^
3	2-Heptenal	57266-86-1	-	10.89 ± 0.43 ^a^	-	9.04 ± 0.42 ^b^
4	(E)-2-Octenal	2548-87-0	-	6.86 ± 0.06 ^c^	9.88 ± 0.12 ^b^	11.68 ± 0.13 ^a^
5	Trans-2-Decenal	3913-81-3	-	3.40 ± 0.08 ^c^	4.36 ± 0.11 ^b^	6.70 ± 0.24 ^a^
6	Nonanal	124-19-6	16.26 ± 0.30 ^c^	17.26 ± 0.34 ^c^	22.87 ± 0.48 ^b^	27.39 ± 0.17 ^a^
7	Benzaldehyde	100-52-7	6.16 ± 0.18 ^b^	7.98 ± 0.17 ^a^	8.03 ± 0.22 ^a^	8.23 ± 0.21 ^a^
	**Total**		74.92 ± 0.79 ^d^	136.75 ± 1.35 ^c^	165.65 ± 1.13 ^b^	199.90 ± 2.36 ^a^
	**Alcohols**					
8	1-Hexanol	111-27-3	5.73 ± 0.22 ^d^	8.07 ± 0.20 ^c^	10.14 ± 0.15 ^b^	12.35 ± 0.26 ^a^
9	1-Octen-3-ol	3391-86-4	5.25 ± 0.09 ^d^	18.51 ± 0.28 ^a^	15.73 ± 0.15 ^b^	7.44 ± 0.17 ^c^
10	Linalool	78-70-6	8.62 ± 0.23 ^c^	9.59 ± 0.28 ^b^	10.32 ± 0.15 ^a^	10.83 ± 0.17 ^a^
11	(-)-Terpinen-4-ol	20126-76-5	51.83 ± 0.92 ^d^	61.58 ± 0.72 ^c^	67.14 ± 0.52 ^b^	70.67 ± 1.29 ^a^
12	α-Terpineol	98-55-5	11.67 ± 0.60 ^a^	9.97 ± 0.32 ^b^	9.08 ± 0.28 ^b^	8.94 ± 0.13 ^b^
	**Total**		83.10 ± 1.24 ^c^	107.72 ± 0.75 ^b^	112.40 ± 0.84 ^a^	110.23 ± 1.12 ^ab^
	**Terpenes**					
13	(1S)-(1)-β-Pinene	18172-67-3	7.52 ± 0.24 ^c^	9.30 ± 0.15 ^b^	10.49 ± 0.19 ^a^	-
14	3-Carene	13466-78-9	19.54 ± 0.33 ^c^	21.00 ± 0.61 ^c^	27.03 ± 0.30 ^b^	32.75 ± 0.82 ^a^
15	4-Carene	29050-33-7	11.57 ± 0.18 ^b^	13.39 ± 0.28 ^a^	13.50 ± 0.23 ^a^	13.91 ± 0.20 ^a^
16	(+)-Dipentene	5989-27-5	34.16 ± 0.54 ^c^	24.61 ± 0.42 ^d^	42.65 ± 0.63 ^b^	49.52 ± 1.00 ^a^
17	β-Phellandrene	555-10-2	16.93 ± 0.20 ^c^	15.92 ± 0.35 ^d^	17.84 ± 0.20 ^b^	19.05 ± 0.24 ^a^
18	γ-Terpinene	99-85-4	53.45 ± 0.27 ^c^	64.79 ± 0.29 ^a^	63.74 ± 0.24 ^b^	63.04 ± 0.35 ^b^
19	2-Carene	554-61-0	18.89 ± 0.21 ^a^	18.54 ± 0.27 ^a^	17.34 ± 0.03 ^b^	17.54 ± 0.27 ^b^
20	α-Copaene	3856-25-5	15.23 ± 0.06 ^d^	16.15 ± 0.21 ^c^	17.54 ± 0.26 ^b^	18.50 ± 0.18 ^a^
21	(+)-7-epi-sesquithujene	159407-35-9	6.91 ± 0.05 ^a^	3.59 ± 0.30 ^b^	-	-
22	β-Sesquiphellandrene	20307-83-9	5.60 ± 0.07 ^a^	3.99 ± 0.12 ^b^	-	-
23	α-Curcumene	644-30-4	9.28 ± 0.20 ^c^	8.27 ± 0.14 ^d^	10.73 ± 0.27 ^b^	11.32 ± 0.11 ^a^
24	β-Bisabolene	495-61-4	10.01 ± 0.27 ^a^	8.32 ± 0.12 ^b^	6.92 ± 0.06 ^c^	6.66 ± 0.21 ^c^
25	β-Pinene	127-91-3	13.69 ± 0.14 ^c^	14.9 ± 0.19 ^b^	15.81 ± 0.41 ^a^	16.08 ± 0.27 ^a^
26	α-Terpinene	99-86-5	9.36 ± 0.26 ^d^	18.52 ± 0.27 ^c^	20.56 ± 0.19 ^b^	24.19 ± 0.24 ^a^
27	Anethole	104-46-1	11.39 ± 0.23 ^c^	11.43 ± 0.17 ^c^	12.67 ± 0.18 ^b^	13.41 ± 0.25 ^a^
	**Total**		243.52 ± 1.65 ^d^	252.73 ± 1.01 ^c^	276.80 ± 0.74 ^b^	285.97 ± 1.59 ^a^
	**Aromatic hydrocarbons**					
28	m-Cymene	535-77-3	-	80.32 ± 0.56 ^a^	-	60.44 ± 0.60 ^b^
29	Diethylbenzene	135-01-3	-	60.54 ± 0.63 ^a^	45.82 ± 0.33 ^b^	40.22 ± 0.20 ^c^
30	1,4-Diethyl-benzene	105-05-5	-	-	99.90 ± 0.52 ^b^	111.04 ± 1.39 ^a^
31	1,3,5-Triethylbenzene	102-25-0	-	44.74 ± 0.21 ^c^	46.27 ± 0.20 ^b^	50.50 ± 0.28 ^a^
32	Benzene, 1-methyl-4-(1-methylpropyl)-	1595-16-0	-	55.69 ± 0.76 ^b^	59.31 ± 0.38 ^a^	-
33	Benzene, 1,3-diethyl-5-methyl-	2050-24-0	-	42.06 ± 0.59 ^c^	57.43 ± 0.30 ^b^	62.68 ± 0.27 ^a^
34	Phenylpentane	538-68-1	-	12.28 ± 0.13 ^b^	14.99 ± 0.17 ^a^	15.72 ± 0.46 ^a^
35	1-Allyl-2-methylbenzene	1587-04-8	-	44.17 ± 0.34 ^c^	59.45 ± 0.43 ^b^	97.36 ± 0.58 ^a^
36	o-Cymene	527-84-4	40.82 ± 0.11 ^a^	38.12 ± 0.21 ^b^	36.09 ± 0.14 ^c^	35.26 ± 0.19 ^d^
37	2,6-Dimethyldecalin	1618-22-0	-	94.14 ± 1.01 ^b^	99.96 ± 0.57 ^a^	-
38	1,4-Dihydronaphthalene	612-17-9	-	-	12.92 ± 0.26 ^b^	14.85 ± 0.28 ^a^
39	2-Methylindene	2177-47-1	-	9.45 ± 0.18 ^b^	11.56 ± 0.21 ^a^	-
40	Elemicin	487-11-6	8.55 ± 0.17 ^b^	5.76 ± 0.17 ^c^	8.16 ± 0.18 ^b^	11.77 ± 0.27 ^a^
41	2-Pentylfuran	3777-69-3	6.40 ± 0.20 ^b^	-	-	10.29 ± 0.13 ^a^
	**Total**		55.77 ± 0.08 ^d^	487.26 ± 1.16 ^c^	551.87 ± 2.16 ^a^	510.13 ± 2.11 ^b^
	**Acids**					
42	Hexanoic acid	142-62-1	2.47 ± 0.16 ^c^	2.52 ± 0.13 ^c^	4.95 ± 0.34 ^b^	6.96 ± 0.26 ^a^
43	Palmitic acid	57-10-3	7.78 ± 0.11 ^c^	11.91 ± 0.30 ^b^	12.26 ± 0.28 ^b^	15.12 ± 0.35 ^a^
44	Nonanoic acid	112-05-0	-	-	9.31 ± 0.17 ^b^	10.49 ± 0.18 ^a^
	**Total**		10.25 ± 0.25 ^d^	14.44 ± 0.41 ^c^	26.52 ± 0.77 ^b^	32.56 ± 0.35 ^a^
	**Esters**					
45	Butyl acetate	123-86-4	9.30 ± 0.05 ^b^	11.72 ± 0.47 ^a^	-	-
46	Butyl sorbate	7367-78-4	-	-	4.76 ± 0.20 ^b^	15.63 ± 0.35 ^a^
	**Total**		9.30 ± 0.05 ^c^	11.72 ± 0.47 ^b^	4.76 ± 0.20 ^d^	15.63 ± 0.35 ^a^
	**Others**					
47	Cis-2-Phenyl-2-butene	768-00-3	-	31.49 ± 0.30 ^b^	35.27 ± 0.34 ^a^	-
48	2,2,4,6,6-Pentamethylheptane	13475-82-6	-	68.95 ± 0.31 ^b^	94.97 ± 0.78 ^a^	-
49	Safrole	94-59-7	12.39 ± 0.23 ^d^	14.58 ± 0.17 ^c^	17.54 ± 0.24 ^b^	18.92 ± 0.16 ^a^
50	Myristicin	607-91-0	12.81 ± 0.30 ^c^	12.67 ± 0.12 ^c^	17.75 ± 0.29 ^b^	23.05 ± 0.56 ^a^
	**Total**		25.20 ± 0.24 ^d^	127.69 ± 0.19 ^b^	165.52 ± 1.31 ^a^	41.97 ± 0.40 ^c^

The contents of volatile compounds are referring to an approximate quantitation that was relative to the concentration of the internal standard. Values are expressed as the mean ± standard error (SE). Different lowercase letters (a–d) in the same row indicate significant differences (*p* < 0.05). “-” indicates that the corresponding volatile compounds were not detected in the samples.

**Table 7:** Approximate odor activity values (OAV) of volatile compounds of frankfurters prepared with 100% lard or lard replaced with 25%, 50% or 100% purified glycerolized lard (PGL).

No.	Volatile Compounds	Odor Threshold (μg/kg in Water)	100% Lard	25% PGL	50% PGL	100% PGL
1	Hexanal	5	9.27	16.65	22.32	24.98
2	Octanal	0.7	8.77	10.13	12.74	17.06
4	(E)-2-Octenal	40	-	<1	<1	<1
6	Nonanal	1	16.26	17.26	22.87	27.39
7	Benzaldehyde	350	<1	<1	<1	<1
8	1-Hexanol	5.6	1.02	1.44	1.81	2.21
9	1-Octen-3-ol	1	5.25	18.51	15.73	7.44
10	Linalool	6	1.44	1.6	1.72	1.81
12	α-Terpineol	1200	<1	<1	<1	<1
13	(1S)-(1)-β-Pinene	4160	<1	<1	<1	-
14	3-Carene	770	<1	<1	<1	<1
16	(+)-Dipentene	34	1	<1	1.25	1.46
17	β-Phellandrene	36	<1	<1	<1	<1
18	γ-Terpinene	3260	<1	<1	<1	<1
25	β-Pinene	140	<1	<1	<1	<1
26	α-Terpinene	41	<1	<1	<1	<1
27	Anethole	50	<1	<1	<1	<1
28	m-Cymene	800	-	<1	-	<1
31	1,3,5-Triethylbenzene	170	-	<1	<1	<1
41	2-Pentylfuran	14.5	<1	-	-	<1
42	Hexanoic acid	40	<1	<1	<1	<1
44	Nonanoic acid	3000	-	-	<1	<1
49	Safrole	160	<1	<1	<1	<1
50	Myristicin	30	<1	<1	<1	<1

## Data Availability

The data presented in this study are available within the article.
